# Insecticide and Repellent Mixture Pour-On Protects Cattle against Animal Trypanosomosis

**DOI:** 10.1371/journal.pntd.0005248

**Published:** 2016-12-27

**Authors:** Geoffrey Gimonneau, Yaya Alioum, Mamoudou Abdoulmoumini, Andre Zoli, Bylah Cene, Hassane Adakal, Jérémy Bouyer

**Affiliations:** 1 CIRAD, UMR INTERTRYP, F-34398, Montpellier, France; 2 Centre International de Recherche-développement sur l’Élevage en Zone Subhumide, BP 454, Bobo-Dioulasso, Burkina Faso; 3 University of Ngaoundéré, School of Veterinary Medicine and Sciences, Department of Parasitology and Parasitological Diseases, Ngaoundéré, Cameroon; 4 Université Dan Dicko Dankoulodo de Maradi, Département des Sciences et Techniques de l’Elevage (FASE/DSTE), BP 465 Maradi, Niger; 5 CIRAD, UMR CMAEE, F-34398 Montpellier, France; Makerere University, UGANDA

## Abstract

**Background:**

African animal trypanosomosis (AAT), transmitted by tsetse flies and tick-borne diseases are the main constraints to livestock production in sub-Saharan Africa. Vector control methods such as pour-on offer individual protection against ticks but not against tsetse so far, for which protection has always been communal, through a reduction of their density. The latter requires the treatment of a large part of the herd in a given landscape and is not instantaneous.

**Methodology/Principal Findings:**

Two prospective surveys were conducted to evaluate the efficacy and persistence of a pour-on formulation composed of cypermetrhin, chlorpyrifos, piperonyl butoxid and citronella (Vectoclor, CEVA Santé Animal). In experimental conditions, tsetse flies were exposed to treated and control cattle. Flies knockdown and engorgement rates were determined and the product persistence was assessed as the time for these parameters to drop below 50% (T50). T50 was 37 days (95%CI: [33–41] days) and 46 days (95%CI: [39–56] days) for the knockdown and engorgement rates respectively. In field conditions, two cattle herds were monitored following a case-control experimental design, in the Adamaoua region of Cameroon. One herd was treated once with Vectoclor pour-on (treated group) and the second used as a control group (not treated). Ticks infestation rate, trypanosomosis prevalence and packed-cell volume were measured over the two months following treatment. The treatment was highly effective against ticks with a complete elimination three days after application in the treated group. Trypanosomosis prevalence was also significantly reduced during the study (by 4, *P*<0.001) and PCV of the treated group increased significantly in the same time (*P*<0.001), contrary to the control group.

**Conclusions/Significance:**

The protection of this new pour-on against tsetse bites and trypanosomosis is demonstrated here for the first time. Moreover, this insecticide and repellent mixture offer a longer persistence of the efficacy against both tsetse and ticks than similar products currently on the market. It offers a great new opportunity for an integrated AAT control strategy including the treatment of residual cases with trypanocides. It might also allow controlling the spread of resistance against these trypanocides.

## Author Summary

In sub-Saharan Africa, tsetse and tick borne disease are the main constraints to livestock production. Providing farmers effective products to control animal pests is a challenging task in the context of increasing resistance to insecticide in many vectors and reduction of available insecticide molecules. Moreover, the spread of invasive species of high economic importance such as the tick *Rhipicephalus microplus* stress the development of new tools. In this study, we evaluated the protective effect of a new pour-on formulation against tsetse, trypanosomosis and ticks in experimental and field trials. This product based on a mix of two insecticides, a repellent and a synergist prove to be very effective with an immediate effect on ticks and tsetse and low treatment frequency to maintain a low ticks infestation and trypanosomosis prevalence. This new insecticide formulation represents an important innovation in the field of vector control, offering a partial individual protection in addition to a collective control method against trypanosomosis vectors and ticks.

## Introduction

Ticks and tsetse are the main vectors of diseases of economic importance to the livestock industry in Africa [1]. The economic cost of ticks borne diseases, mainly babesiosis, cowdriosis and anaplasmosis has been estimated between US$ 13.9 and 18.7 billion [2]. Ticks also cause direct injuries associated to strong economical loses, especially *Amblyomma variegatum* [3]. Also, African animal trypanosomosis (AAT) is one of the major constraints to livestock production in many sub-Saharan African countries infested with tsetse flies [4]. The economic cost of AAT in Africa has been estimated at USD 4.75 billion per year [5]. Together, ticks and tsetse are major constraints to the development and intensification of cattle rearing systems in Africa.

So far, trypanosomosis is mainly controlled through prophylactic and curative drugs. This approach is no longer sustainable, because of the increasing development of drug resistance [6]. Alternatively, different vector control methods are available to reduce infestation impact such as insecticide-treated targets (ITT) for tsetse (e.g. traps and screens impregnated with insecticides) and insecticide-treated cattle (ITC) for ticks and tsetse (e.g. pour-on, spray and dip) [7]. ITT are highly effective to control tsetse and are probably the most cost-effective technique but are difficult to be maintained by farmers since they often consider insecticide traps and targets as public goods which are generally not maintained after the end of government programs [8]. ITC act as a very attractive lethal trap for tsetse due to their odor, movement and size and have also a wider spectrum of action, especially against ticks, stomoxines, tabanids and in some cases, mosquitoes [9–11].

Pour-ons are easy and convenient to use when compared with dipping and spraying technics. There are ready-to-use liquid formulations applied along the cattle backline. They are also more costly than other insecticide treatment strategies but do not require to be mixed with water, a great advantage in some situations, particularly in the case of transhumant herds. As many other control means, limits in pour-ons use are their persistence and efficiency, mainly against insecticide resistant species, such as the Asian cattle tick *Rhipicephalus microplus* that is currently invading Africa [12]. This species is resistant to most of acaricides families such as organophosphates, pyrethroids, amitraze and ivermectin [13, 14] leading to major economic losses to cattle producers through direct and indirect effects as blood sucking and transmission of infectious disease agents. Therefore, there is a need to develop new vector control tools such as more persistent and efficient formulations against these important vectors that are ticks and tsetse flies.

CEVA-Santé Animale recently developed a new pour-on product, so-called Vectoclor, which is composed of two insecticides, cypermethrin (5g/l) and chlorpyrifos (7g/l), mixed with piperonyl butoxide (5g/l), a pyrethroids synergist, and citronella that acts as a repellent (0.5g/l). This mixture has been previously tested in South America and seemed to be the most efficient formulation against the multi-resistant tick *R*. *microplus* [15]. Indeed, one of the most promising strategies to prevent or delay the development of resistance in vectors is the use of products that combine at least two molecules having unrelated modes of action [16]. Theoretically, pests that are resistant to one insecticide should be killed by the other component. This product is encountering a great commercial success in Africa but persistence and efficiency of this new formulation has not been yet tested against tsetse flies and ticks in Africa. Therefore, the aim of this study was to evaluate the protective effect of this new product against trypanosomosis vectors and ticks in experimental and field trials.

## Materials and Methods

### Experiment 1: experimental evaluation of Vectoclor against tsetse

This first experiment was conducted from August to November 2009 at the Centre International de Recherche-Développement sur l’Elevage en zone Subhumide (CIRDES), Burkina Faso, in an experimental stable covered by a metal screen. Six crossbred cattle (Zebu/Baoule, the most frequently encountered cattle breed in Burkina Faso) of comparable size (150-225kg) were used for these trials. Before the study and for each repeat, each animal was presented before treatment to tsetse flies and the engorgment rate was measured to assess any differences of attractiveness or defense reactions. Cattle were treated either with Vectoclor pour-on or with Cypertraz pour-on, the latter considered as positive control. Cypertraz formulation is also based on a mixture of two insecticides (amitraze, 17.5 g/l and cypermethrin 15 g / l) without any repellent or synergist. The effect of amitraze could be neglected against tsetse flies because it has been highlighted to be ineffective and none-persistent (less than one week). For that reason, it has been used as control in previous studies [17]. Two replicates were conducted starting on 23 August and 27 October 2009, and animals used as controls (not receiving any treatment) in the first trial were treated in the second. Therefore, for each replicate, four animals were used: 1 treated with Vectoclor pour-on, 1 treated with Cypertraz, and 2 untreated control cattle. For each replicate, cattle were exposed 10 to 12 successive times to tsetse flies after treatment. The Table 1 shows the number of exposures and the type of treatment carried out on each animal. The animals were exposed to sunlight for 3 hours and watered entirely with 50 liters of water every other day to mimic natural conditions in the rainy season. All along the study, cattle were housed separately in a stable.

**Table 1:** Number of exposure sessions to tsetse flies for each animal by treatment. Two cattle (B29 and B36) were used alternatively as negative controls during the first replication and as treated animals during the second to reduce the importance of any potential individual effect.

ID number	Vectoclor	Cypertraz (positive control)	Control (negative control)
B25	12		
B28		12	
B29		10	12
B36	12		12
BB1683			12
BB5386			10

Trials started the day after the treatment of cattle with Vectoclor and Cypertraz. Tsetse flies were exposed to treated-cattle every 5 days and the experiment ended when tsetse flies knockdown rate was below 50% for 5 successive sessions. The stables were routinely washed after each session and humidified 1 hour before each release to ensure a humidity rate above 75%. The temperature and humidity were measured during all release sessions. To assess the impact of insecticide treatments on flies, 100 males of *Glossina palpalis gambiensis* were released on an animal placed in a stable covered with a metal net for two hours, from 8:00 to 10:00 AM. After exposure, tsetse flies were collected and classified according to their engorgement state (blood feed or not) and knockdown state. Tsetse flies that knocked-down after 2 hours were considered dead because it is assumed from previous work that the majority of paralyzed tsetse flies will perish in field conditions [18].

### Experiment 2: field evaluation of Vectoclor against ticks and animal trypanosomosis

#### Study site

The study was conducted in the village of Sarkimata (Latitude: 7.883273, Longitude: 12.566496), located in the department of Faro and Deo Division, in the Adamaoua region of Cameroon. The region is characterized by a Sudano-Guinean climate with two main seasons: a rainy season from April to October and a dry season from November to March. Mean annual rainfall is 1500 mm for 120 to 150 days of rains per year and average temperatures range between 23–25°C [19]. The vegetation in this area is diversified, with two main groups of bushlands dominated by *Daniellia oliveri* and *Lophira lanceolata* covering the Plateau of Adamaoua [20]. Rivers present in this area harbor gallery forests infested by two tsetse species: *Glossina morsitans submorsitans* and *Glossina tachinoides* [21]. Up to 7 tick species are also present with *Rhipicephalus (Boophilus) decoloratus* and *Amblyomma variegatum* as principal abundant species [22]. The study was done in the rainy season, from May 10^th^ to July 24^th^ 2014.

#### Animal treatment

Two zebu herds of 30 animals (trypanosensible crossbreed of zebu/goudali and zebu/ Bororo) were selected in the ranch of Sarkimata, and were identified with numbered ear tags (1 to 30). All animals were of similar weight ranging between 300 and 400kg and were all bulls. One week before the start of the experiment, all animals were examined for trypanosomes by the buffy coat method. All positive animals were treated one week before treatment with VERIBEN (diminazene aceturate, 7mg/kg).

One herd was used as a control in the survey (i.e. no insecticide treatment) and the second was treated with an application of pour-on solution of Vectoclor (10ml/100kg live weight) along the backline of cattle. The two groups of cattle grazed in the same site and shared the same drinking points.

#### Tick count and infestation assessment

A preliminary entomological survey was conducted on twenty animals (independently of the two herds of 30 animals) before the beginning of the study in May 2014 in order to determine tick species present in the area. For the main survey, animals were randomly selected for follow-up and ticks were collected over the whole body of the chosen animals: 18 animals were selected in the cattle control group and 19 in the treated-group. Ticks were then conserved in 70% ethanol and identified to the species level under a stereomicroscope using the morphological keys for the Afrotropical region [23].

Ticks infestation in the two survey herds was determined before and all along the study at different time after treatment: day 1, 3, 8, 18, 25 and 32 [24]. Ticks were counted on the left half of the chosen animals (previously tethered), and the total number of ticks per animal was then derived by multiplying by two. Vectoclor efficacy on ticks’ infestation was determined by comparing the number of ticks alive on animals before and after the treatment.

#### Parasitological prevalence

In order to monitor the trypanosomoses prevalence before and during the 60 days of the study, all animals were blood-sampled by a veterinary at 9 different times (days -7, 0, 7, 17, 24, 31, 45, 52 and 60). Five milliliter of jugular blood was collected from each cattle into heparinized tubes and stored at 4°C in a cooler. Microtubes of blood were centrifuged at 8000 spins min-1 during 4 minutes, just after blood uptake. Packed-cell volume (PCV) was then measured and the buffy coat method was applied: thin smears were done with buffy coats and examined for trypanosome detection with a microscope (40x10). Trypanosome species were identified morphologically. PCV measurements and microscope examination were carried out on the field. During the monitoring period, positive animals to the buffy coat method and animals presenting a PCV below 15 associated with some signs of the disease were treated with Veriben (Diminazene aceturate 7mg/kg).

### Statistical analysis

#### Experimental trial

Data from experiment 1 were analyzed using a beta binomial model as described previously [8, 25]. The dependent variable was either the tsetse knockdown (KD) rate after exposure to treated animals or the rate of engorgement flies. The explanatory variables were: the treatment type, the processing date, the duration since impregnation expressed in days and their interactions. The best model was selected on the basis of the lowest corrected Akaike information criterion (AICc), and the significance of fixed effects was tested using the likelihood test ratio [26, 27]. This analysis allowed assessment of times after treatment for which the knockdown and engorgement rates dropped below 50% (T50).

#### Field trial

Ticks abundance and PCV data from experiment 2 were analyzed with a linear mixed-effects model (LMER) [28, 29]. The trypanosomes prevalence (all species together) was analyzed with a generalized linear mixed binomial model fit by maximum likelihood. The cattle individuals were represented by a random effect and the fixed effects were treatment (categorical variable, either pour-on or control), time after treatment and their interaction. The best model was selected on the basis of the lowest corrected Akaike information criterion (AICc), and the significance of fixed effects was tested using the likelihood test ratio [26, 27]. The R (version 3.2.1) software was used for data analysis [30].

### Ethical statement

The protocol strictly adhered to the guidelines of the national ethical committee and general direction of veterinary services of the Ministry of Animal Resources of Burkina Faso. Animals used as baits were the property of CIRDES and were no longer exposed to tsetse bites than other animals in the natural environment. They also received veterinary care as much as required during the whole experiment.

Experiment 2 was approved by the Department of Parasitology and Parasitological Diseases of the School of Veterinary Medicine and Sciences of the University of Ngaoundere, Cameroon, which give necessary permissions. Cattle belonged to a local farmer and informed consent was obtained from him before insecticide treatments and blood sampling were carried out in his herd.

## Results

### Experiment 1

Cattle attractiveness was similar between the animals used in each repetition. For the first trial, the engorgement rate before any treatment was 0.58 (SD 0.03) and similar between animals (X-squared = 1.3422, df = 3, p-value = 0.7191). For the second one, it was 0.89 (SD 0.04) (X-squared = 4.2749, df = 3, p-value = 0.2333).

**Fig 1:**
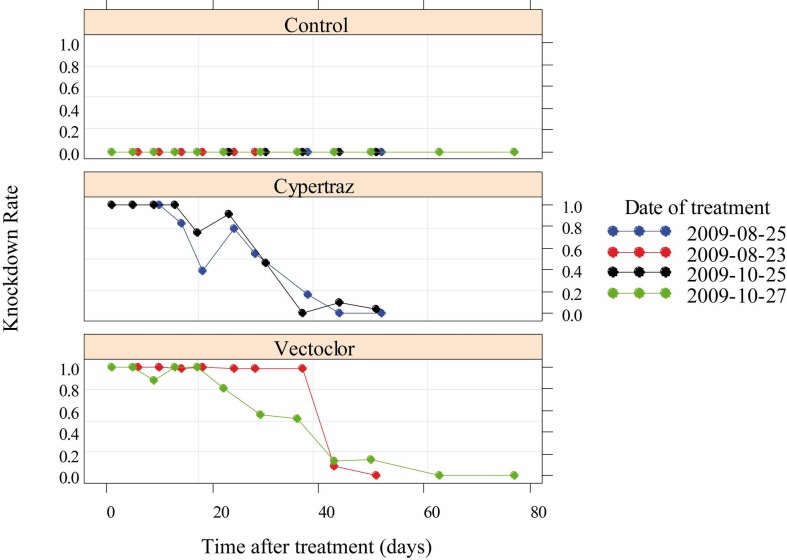
Knockdown rate of *G*. *p*. *gambiensis* since the initial treatment (in days).

The best model (lowest AICc) selected for the knockdown rate analysis retained the type of treatment and the duration since impregnation as variables that fitted well the data with no significant differences between the two trials session (*P* = 0.98). Fig 1 presents the KD rates of flies exposed to control or treated cattle (Vectoclor and Cypertraz). The Vectoclor insecticide activity was longer than Cypertraz. The time taken to knockdown 50% of the exposed flies was 37 days (95%CI: [33–41] days) for Vectoclor and 28 days (95%CI: [24–32] days) for Cypertraz. No KDR effect was observed in the control all along the experiment.

**Fig 2:**
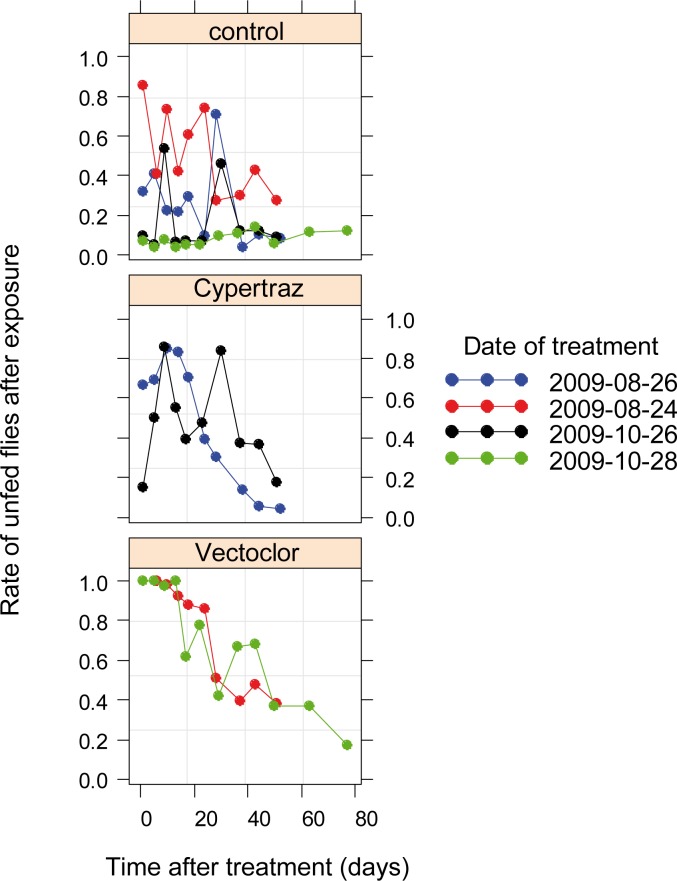
Protective rate against *G*. *p*. *gambiensis* blood meal since the initial treatment (in days).

Results of the tsetse flies engorgment rate model (here considered as proxy of the protective effect of the treatments against tsetse bites) retained the type of treatment and time since impregnation. Data fitted well with no significant differences between the two trials session (*P* = 0.22). Fig 2 presents the protective effects of treatments over time. The Cypertraz showed an irregular protective effect, especially during the second trial session with an average T50 of 21 days (95%CI: [15–28] days) whereas the Vectoclor was more regular and persistent with 46 days (95%CI: [39–56] days).

### Experiment 2

#### Ticks species and infestation rate

The total number of ticks collected during the preliminary entomological survey before the study on 20 animals was 462, with a mean of 23.1 ticks per animal. Three species were found: *Amblyomma variegatum* (382), *Rhipicephalus (Boophilus) annulatus* (52) and *Rhipicephalus (Boophilus) decoloratus* (28) with *A*. *variegatum* representing the major part of the collection (Table 2). Adult stages of *A*. *variegatum* were particularly more numerous during this period of the early rainy season. Few males of the genus *Rhipicephalus (Boophilus)* were collected.

**Table 2:** Number of ticks per species and sex collected during the preliminary entomological survey

Ticks Species						
	*Amblyomma variegatum*		*Rhipicephalus annulatus*		*Rhipicephalus decoloratus*	
	Males	Females	Males	Females	Males	Females
Number of ticks/species/sex	301	81	4	52	0	28
Total number/species	382		52		28	
Species proportion (%)	82.69		11.25		6.06	

**Fig 3:**
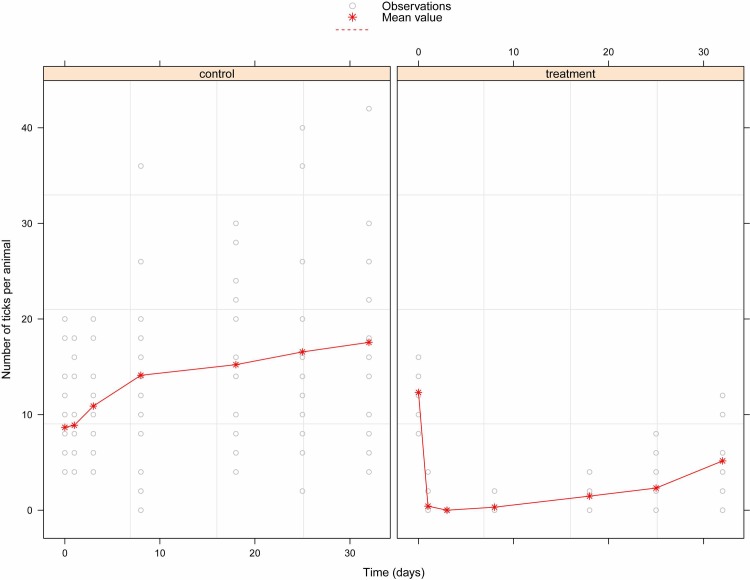
Observed numbers of ticks attached to the control and treated cattle. The treated cattle received a pour-on formulation of Vectoclor at the start of the study. Red stars represent the mean value of ticks per animals and grey dots individual data. Note that some dots are superposed and thus not visible.

With regard to the two herds surveyed, initial infestation was lower in the control herd than in the treated one (*P* = 0.021) with a mean infestation intensity of 8.66 (sd 5.36) and 12.31 (sd 3.48) ticks/animal respectively (Fig 3). After the Vectoclor treatment, the infestation rate decrease quickly in the treated group with no ticks found at day 3. In contrary, infestation rate increased in the control group all along the survey and reached three-fold higher at the end of the study compared to the treated group (mean 17.55 ticks/animals and 5.15 ticks/animals respectively).

The fixed-effect coefficients of the linear mixed-effects model of ticks abundance are shown in Table 3. A strong negative effect of the Vectoclor treatment was observed on ticks abundance in the treated group, thus indicating that ticks abundance was significantly lower in the treated than control herd during all the survey (*P*< 0.001). The protective effect of treatment however reduced with time (*P*<0.001).

**Table 3:** Coefficients of the linear mixed-effects model of ticks infestation for the two groups of cattle in Sarkimata (Adamaoua region, Cameroon).

Coefficient	Estimate	Standard error	t value	p value
Intercept	11.042	0.913	12.099	<0.001
Time	0.195	0.028	6.921	<0.001
Treated group	‐11.736	0.729	‐16.105	<0.001

#### Trypanosomes prevalence and diversity

**Fig 4:**
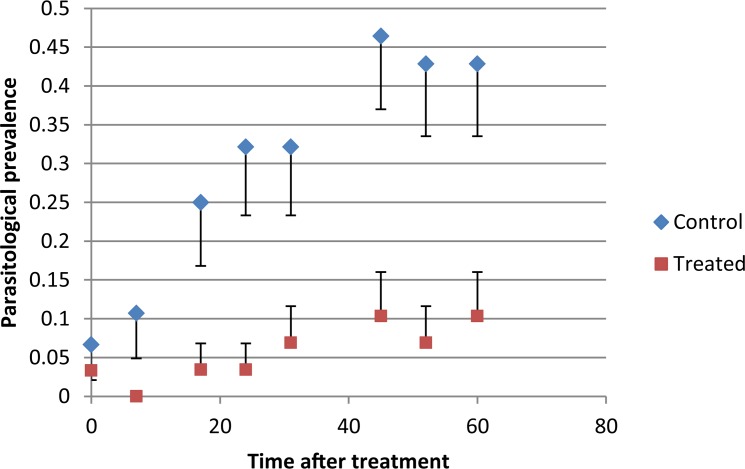
Trypanosomes prevalence of the control and treated cattle. Treated cattle received a pour-on formulation of Vectoclor at the start of the study. Points represent the mean value of trypanosomes prevalence and vertical bars the standard error.

Before the experiment, trypanosomes prevalence in the study area was assessed on the 60 cattle selected and was 27.8% (*Trypanosoma sp*.). The predominant species was *Trypanosoma congolense* (50%) followed by *T*. *vivax* (30%) then *T*. *brucei* sl. (18.6%). Mixed infections (*T*. *congolense* + *T*. *vivax*) represented 1.4% of the positive animals. During the survey, the number of cattle presents in the treated and control groups decreased due to the non-presentation of 2 animals in the control group and 1 animal in the treated group. Changes in trypanosomosis prevalence are displayed in Fig 4. In the control group, the prevalence rate was already high at day 7 and increased throughout the study period to 44.82% at the end of the survey (Fig 4). In the treated group, the prevalence rate remained null until day 7 and rose up to 10% by the end of the study. Overall differences between the control and treated prevalence was strong and significant (*P*<0.001).

The fixed-effects of the generalized linear mixed model fit by maximum likelihood are shown in Table 4. The treatment effect was negative indicating a significantly lower trypanosomosis prevalence in this group (*P*<0.001). The risk of trypanosomes infection was significantly reduced by 4 times in the treated group (*P*<0.001). The protective effect of treatment however reduced with time (*P*<0.001).

**Table 4:** Coefficients of the generalized linear mixed model fit by maximum likelihood of trypanosomosis prevalence for the two groups of cattle in Sarkimata (Adamaoua region, Cameroon).

Coefficient	Estimate	Standard error	*P*
Intercept	-2.577	0.471	<0.001
Time	0.043	0.009	<0.001
Treated group	-2.662	0.408	<0.001

#### Packed-cell volume

**Fig 5:**
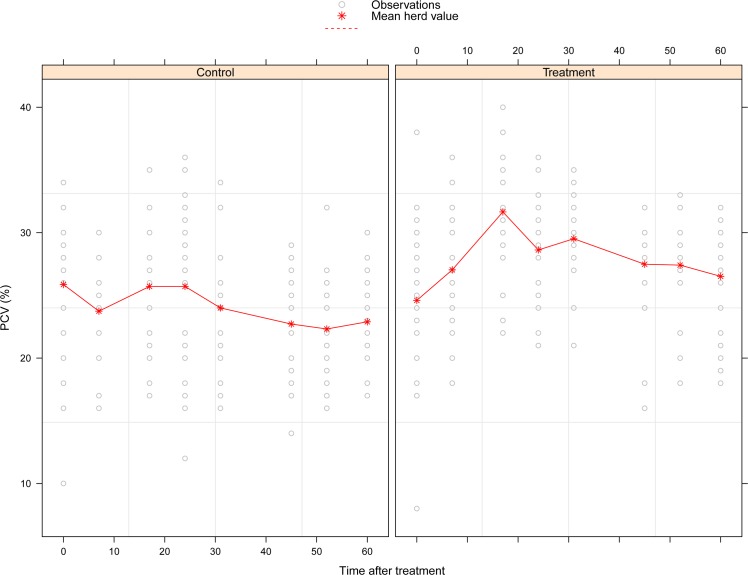
Change in packed-cell volume in Sarkimata (Adamaoua region, Cameroon) according to the treatment category. Red stars represent the mean value of PCV and grey points individual data. Note that some dots are superposed and thus not visible.

PCV data are shown in Fig 5. Mean patterns were different for the control and treated cattle groups. In the former, mean PCV decreased from 25.87% (sd 5.50) at the start of the study to 22.89% (sd 3.58) by the end. For the treated-cattle group, mean PCV showed the reverse evolution with a strong and fast increase, especially during three weeks after the start of the experiment from 24.60% (sd 5.89) to 31.65% (sd 4.49). Thereafter, the mean PCV decreased slowly to 26.51 (sd 4.25) at the end of the study.

The fixed-effect coefficients of the linear mixed-effects model of PCV are shown in Table 5. The treatment effect was positive indicating a significantly higher PCV in this group (*P*<0.001). The protective effect of treatment however reduced with time (*P* = 0.017).

**Table 5:** Coefficients of the linear mixed-effects model of PCV for the two groups of cattle in Sarkimata (Adamaoua region, Cameroon).

Coefficient	Estimate	Standard error	t value	p value
Intercept	24.84188	0.49727	49.96	<0.001
Time	‐0.02478	0.01041	‐2.38	0.017
Treated group	3.66119	0.42406	8.63	<0.001

## Discussion and Conclusion

The objective of this study was to evaluate a new pour-on formulation against trypanosomosis vectors in experimental conditions in Burkina Faso, and ticks and trypanosomosis infection in cattle in Adamaoua region, Cameroon. Comparative results obtained between Vectoclor and Cypertraz highlighted that the first one has a longer protective effect against tsetse bites (i.e. repellency) and a longer insecticide effect. Although Cypertraz protection was acceptable, the observed differences were probably due to the repellent effect of the Vectoclor formulation. In field conditions, Vectoclor application was highly effective against ticks with a complete elimination, three days after application. Trypanosomosis prevalence was reduced by 4 during the study and PCV of the treated group increased in the same time.

The experimental trial compared Vectocolor formulation to Cypertraz with the latter considered as a positive control. These two products are based on a mix of insecticides and share the same synthetic pyrethroid, cypermethrin. Vectoclor also contains a pyrethroid synergist and a repellent and their additive effects resulted in a more effective and persistent formulation than Cypertraz (cypermethrin mixed with amitraze). Nonetheless, these two products present a good protection against tsetse bites in comparison to other epicutaneous treatments [25, 31]. Indeed, the T50 for the tsetse KD rate measured in this study was much higher than previous study conducted under the same conditions at CIRDES for animals entirely sprayed with a 0.005% solution of deltamethrin (T50 3 days, 95%CI = [0–5] days for Vectocid) [25] or with a 0.005% solution of alpha-cypermethrin (T50 3 days, 95%CI = [0–5] days for Dominex) [31]. Results obtained for Cypertraz was comparable to a flumethrin pour-on (1 mg of active ingredient/kg) with a T50 for KD rate of the exposed flies of 28 days (95%C.I. [24–32] days) [32, 33], but the Vectoclor persistence was better (T50, 37 days, 95%CI = [33–41] days). The combination of a repellent to several insecticides in Vectoclor appeared highly interesting since it is currently the most effective and persistent product against tsetse flies. This repellent effect probably had a positive effect on the knockdown rate since flies that cannot feed are more sensitive to the exposure to a given dose of insecticide and thus more likely to die [34, 35].

Field trial of Vectoclor proved that it was very effective against ticks and tsetse. Separately, Vectoclor active ingredients have been proven effectives against tick and tsetse [25, 36] but some resistance was reported, especially against the ticks such as *Rhipicephalus (Boophilus) microplus* [37]. In the study area, three species of ticks have been found: *Amblyomma variegatum*, *Rhipicephalus (Boophilus) annulatus* and *Rhipicephalus (Boophilus) decoloratus*. This result are in accordance to previous studies [22], although species relative abundance was not the same probably due to seasonal effect. Few males of the genus *Boophilus* were collected. It was difficult to collect them due to their small size and location below the females. Although an alarming spread of the Asian cattle tick *R*. *microplus* in West and Central Africa is currently ongoing, this species was not found in our study and a previous one in the same area [22]. Impregnation of cattle with Vectoclor pour-on was efficient to control ticks in field conditions. Indeed, despite a higher initial infestation intensity in the treated herd, ticks were eliminated three days after treatment in the treated herd and one month after treatment, the observed infestation rate remained three times lower than the control herd. Previous studies using different pour-on formulations of pyrethroids or organophosphate reached this low ticks burden 2 to 3 weeks after pour-on treatment [38] and sometimes after repeated applications. This same observation was made for footbath acaricide treatment where repeated applications were needed to reduce ticks infestation near to zero [39].

Moreover, we observed an overall significant difference of cattle trypanosomosis prevalence between the control and treated herds over the two months study period, with no positive animal until day 7 in the treated group, probably due to the important effect of citronella repellent and pyrethroids volatiles against tsetse flies that are highly susceptible [25]. Although the trypanosomosis prevalence observed at the end of the study was higher than the one observed at the beginning (44.8% and 27.8% respectively), these data are in accordance with the annual prevalence of trypanosomosis in this region (55.2%) [40]. This result may probably not reflect the exact parasitological status of cattle because the buffy coat method is not as sensitive as the PCR methods to detect trypanosome infections [41]. However, the significant increase in PCV observed all along the study period in the treated herd suggests that parasite load was significantly reduced, on the opposite to the control herd where PCV slowly decreased. Although entomological data on tsetse apparent density was not available, parasitological results showed that treated cattle experienced a significant reduction in the host vector contact, with a trypanosomosis prevalence rate below 10% in two months. The two main tsetse species present in the Adamaoua region are *G*. *morsitans submorsitans* and *G*. *tachinoides* and both species are susceptible to cypermethrin [25]. However, since the two herds grazed and drank water in the same areas, the observed difference in prevalence was probably due to the partial individual protection observed during the experimental trial than a communal protection linked to a reduction of tsetse densities in the area but this need to be confirmed by field entomological studies. This also could explain why the protective effect was so fast to appear in the treated herd (< 10 days).

This new pour-on formulation presents several advantages especially its immediate effect on ticks and the low treatment frequency needed to maintain a low ticks infestation and trypanosomosis prevalence. In the study area, only one treatment per month is advisable to maintain a tick infestation rate below 10 ticks/animals and a two month frequency seems sufficient to maintain a trypanosomosis prevalence below 10% whereas the parasitological prevalence reached very high levels in the control herd (up to 46%). This is probably the result of combining two insecticide molecules (cypermethrin and chlorpiryphos) with a synergist and a repellent. Combinations are particularly interesting when there is potentiation between the two insecticides as this would make it possible to lower the dosage of each, as demonstrated under laboratory conditions [42]. This is the case for Vectoclor that contained three times less cypermethrin than Cypertraz (i.e 5g/l against 15g/l). Insecticides and repellent mixtures have also been successfully used in public health against mosquitoes that present increasing levels of insecticide resistance, and this combination proved highly effective [43, 44]. This is also the case for the Vectoclor combination that has been shown to be effective against the invasive and multi resistant ticks *Rhipicephalus (Boophilus) microplus* [15, 45]. One drawback of this product could be its cost, especially for farmers from developing countries. Actually, comparing with the Vectoclor emulsifiable concentrate (EC) formulation, the cost of treating one animal with the Vectoclor pour-on formulation is 2.3 times higher. It is noteworthy however that the treatment cost per one animal using Vectoclor EC formulation is only 40% more expensive than an EC formulation containing deltamethrin only and provided by the same manufacturer (Vectocid). This might represent a valuable investment, although the individual protection against trypanosomoses by the Vectoclor EC formulation has still to be confirmed.

In conclusion, the insecticide mixture of Vectoclor was highly effective against AAT and ticks and to our knowledge is the most persistent pour-on on the market. However, this does not mean that the use of Vectoclor will fully prevent cattle trypanosomosis and ticks infestation everywhere, in any conditions, especially in the presence of mechanical vectors such as tabanides. The efficacy of vector control tools are context dependent and their effect could be different according to different vector species or environment [46]. They however represent a necessary tool to combine with trypanocide treatment of the remaining clinical cases within an integrated management vision. The use of Vectoclor pour-on will allow reducing the number of trypanocide treatments and thus the selection pressure which should eventually result in a reduced spread of resistance [47]. According to our results, this new insecticide formulation represents a partial individual protection in addition to a collective control method against trypanosomosis vectors and ticks.

## Supporting Information

S1 Table Database for experimental evaluation of Vectoclor against tsetse in Burkina Faso. (XLS)

S2 Table Database of tick’s infestation for the field evaluation of Vectoclor in Cameroon. (XLSX)

S3 Table Database of trypanosomosis infections for the field evaluation of Vectoclor in Cameroon. (XLSX)

S4 Table Database of packed-cell volume for the field evaluation of Vectoclor in Cameroon. (XLSX)
